# Supplementary figures and images for: Evolutionary history of host trees amplifies the dilution effect of biodiversity on forest pests
Source: PLoS Biol. 2024 Feb 27;22(2):e3002473. doi: 10.1371/journal.pbio.3002473 (PMC10898760; doi:10.1371/journal.pbio.3002473)

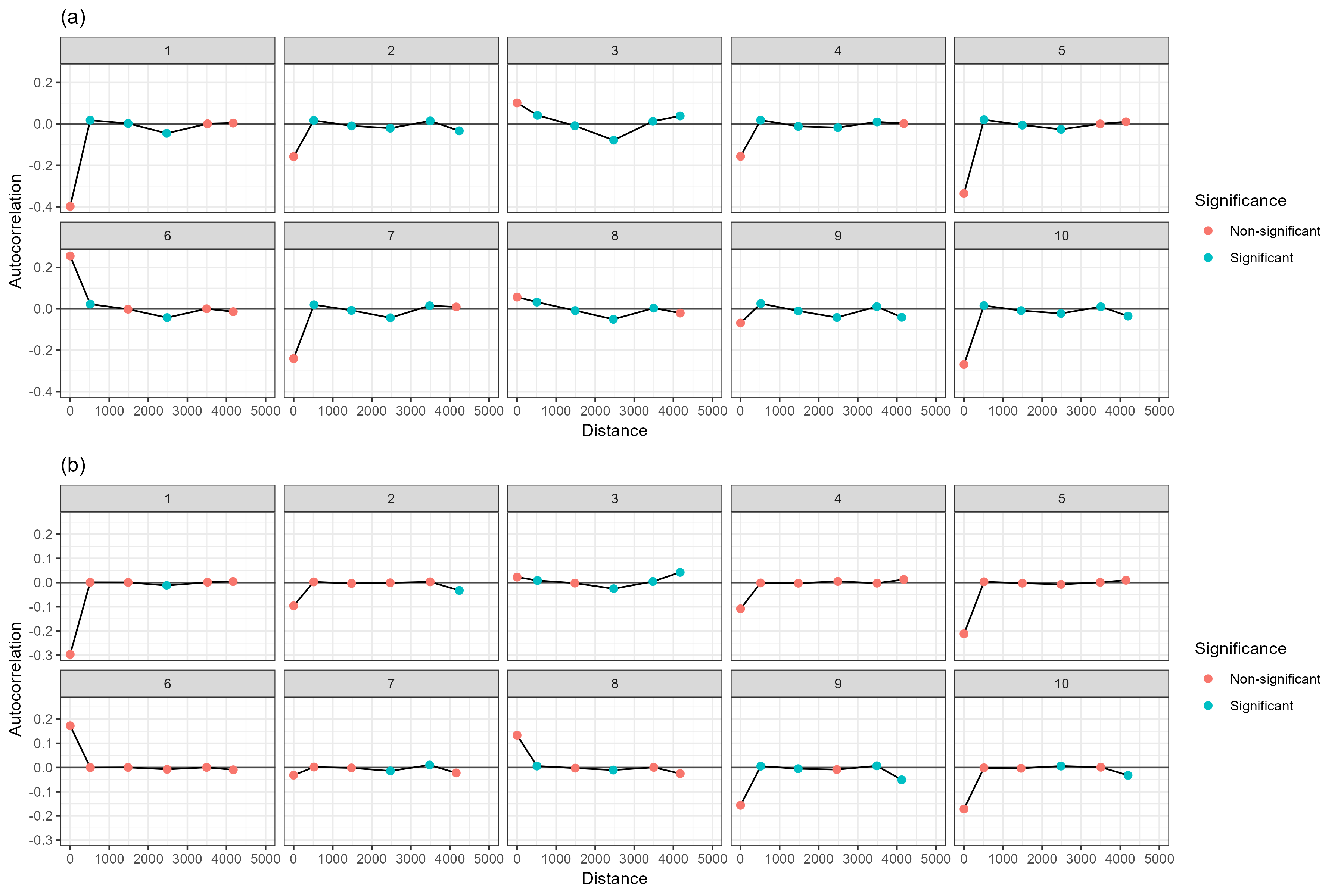

Supplement: S1 Fig — Each panel shows a random draw of 1,000 plots used to calculate spatial autocorrelation. Significance is determined by a two-sided permutation test, and indicates p < 0.025 or p > 0.975. Note autocorrelation is not significant for most distances in (b), indicating that the model is accounting for much of the spatial pattern. Data underlying this figure can be found at doi.org/10.6084/m9.figshare.21317979. (TIFF) [file pbio.3002473.s001.tiff]

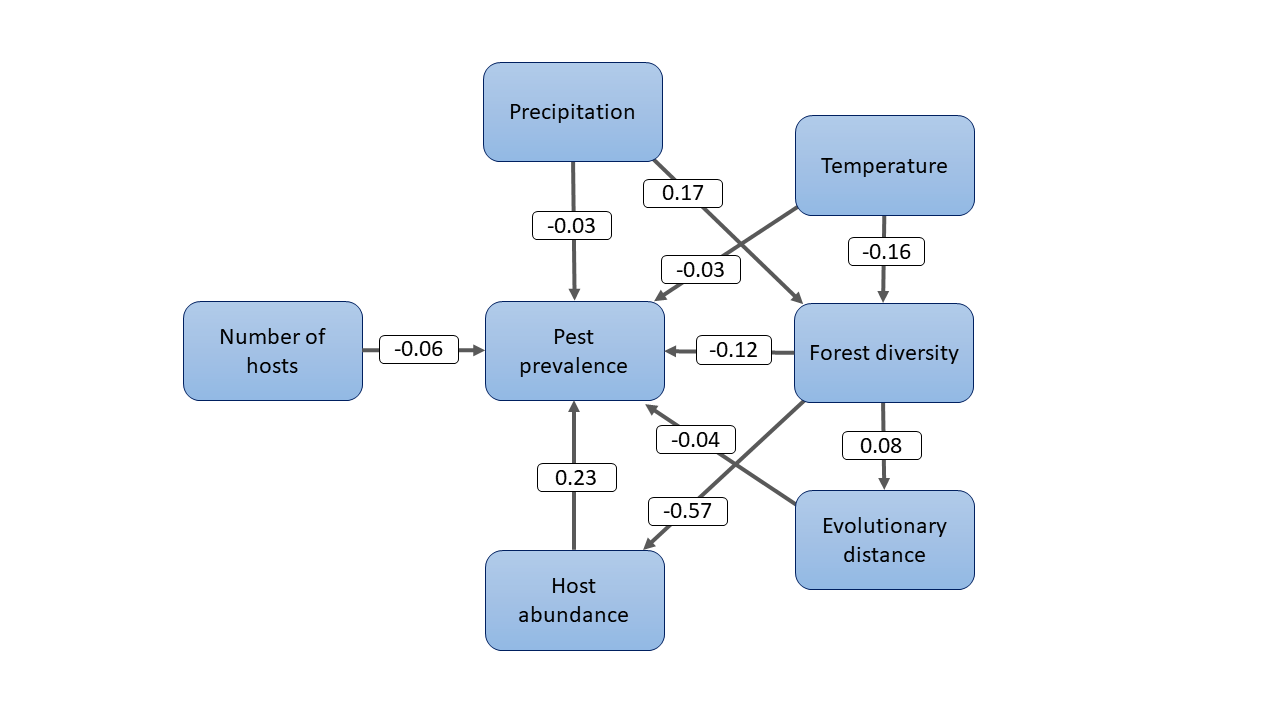

Supplement: S2 Fig — The dataset used to fit this model was identical to the one used for the Bayesian model, but allowed for effects between diversity, temperature and precipitation, and diversity, evolutionary distance, and host abundance. Numbers show standardized effect sizes. Data underlying this figure can be found at doi.org/10.6084/m9.figshare.21317979. (TIF) [file pbio.3002473.s002.TIF]

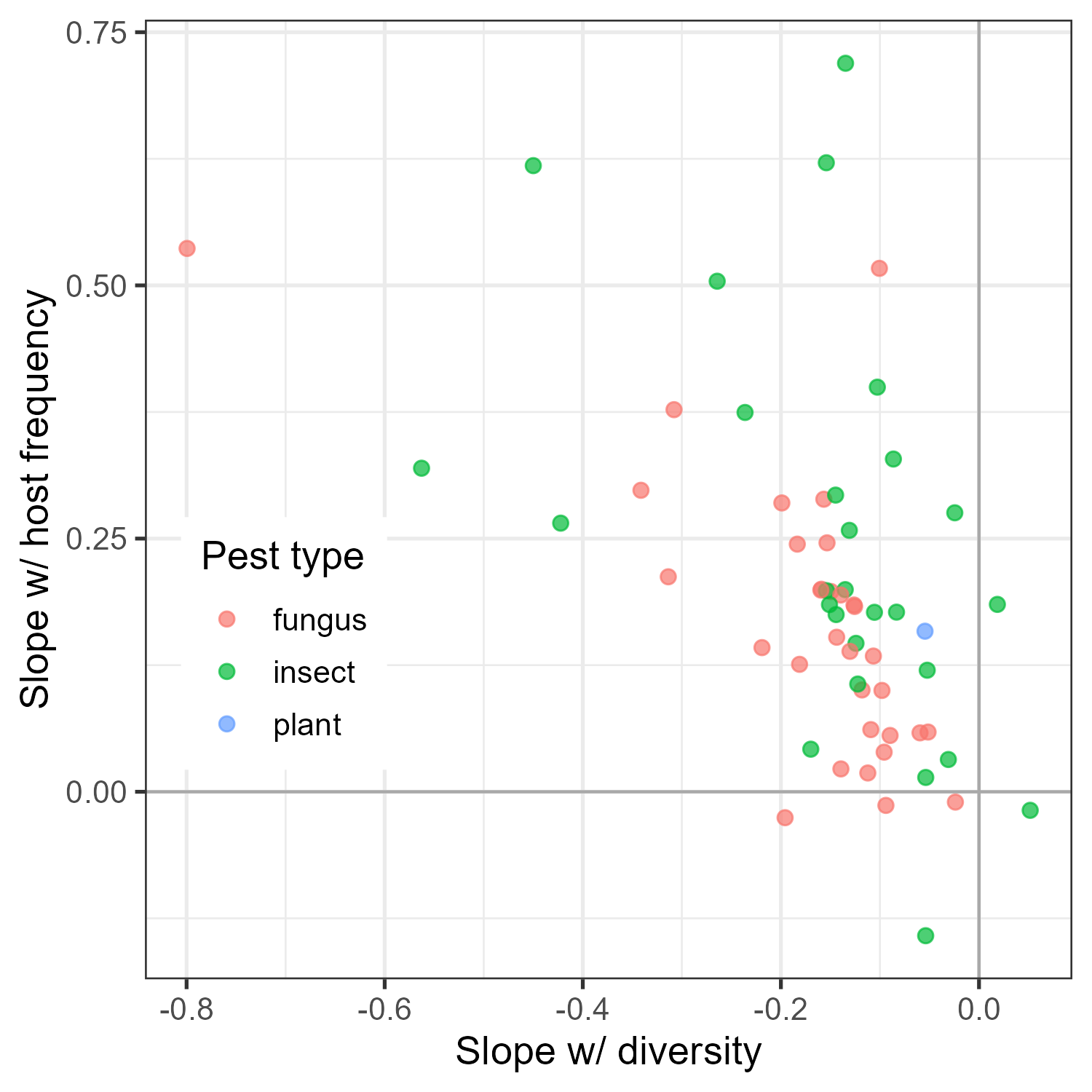

Supplement: S3 Fig — Pests are colored by general pest type; fungi include fungi-like organisms. See also Fig 2 and S4. Data underlying this figure can be found at doi.org/10.6084/m9.figshare.21317979. (TIFF) [file pbio.3002473.s003.tiff]

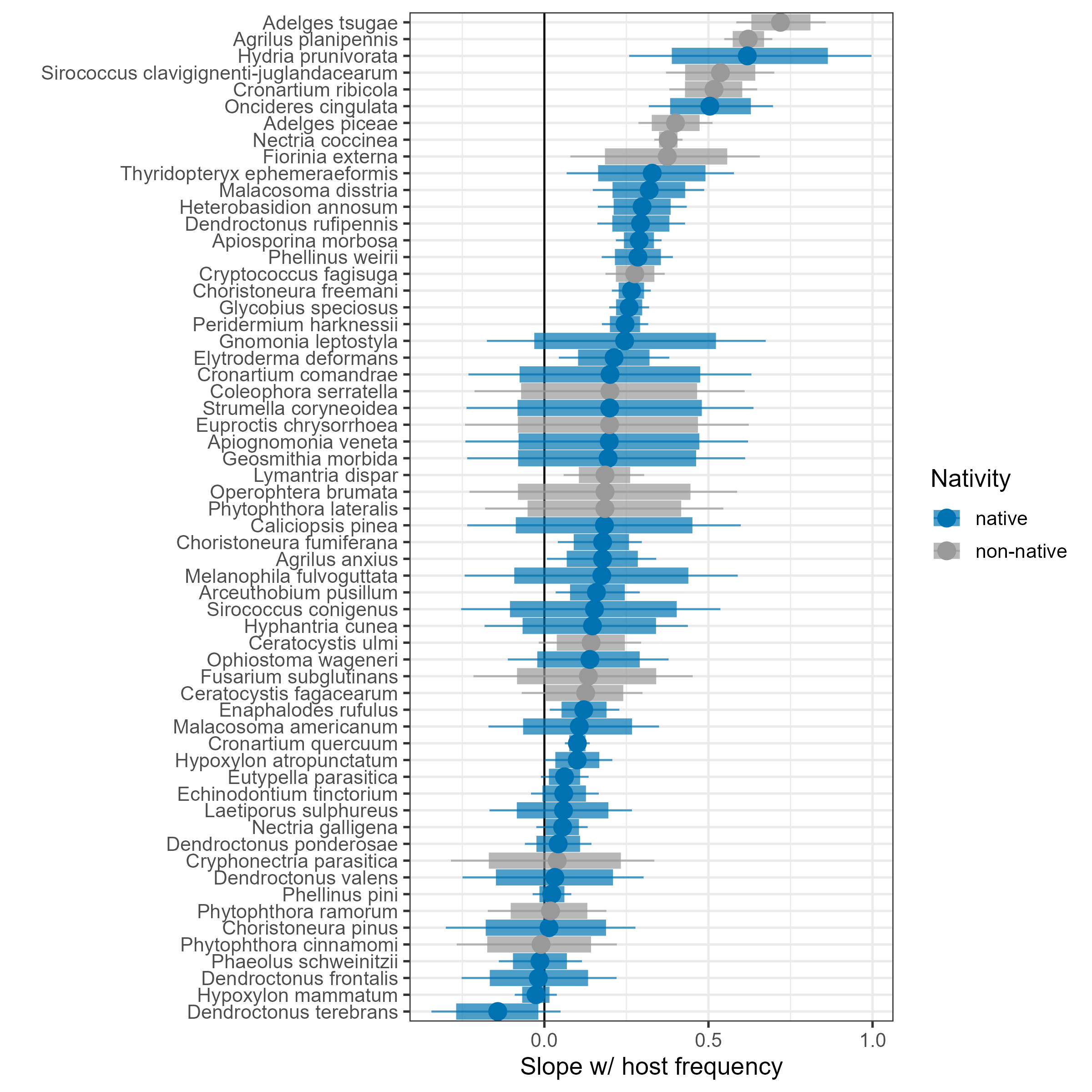

Supplement: S4 Fig — Negative values indicate a tendency towards amplification, and positive values indicate a tendency towards dilution. Points are colored by pest nativity. Points are medians, shaded segments are 80% credible intervals, and thinner lines are 95% intervals. See also Fig 3. Data underlying this figure can be found at doi.org/10.6084/m9.figshare.21317979. (TIFF) [file pbio.3002473.s004.tiff]

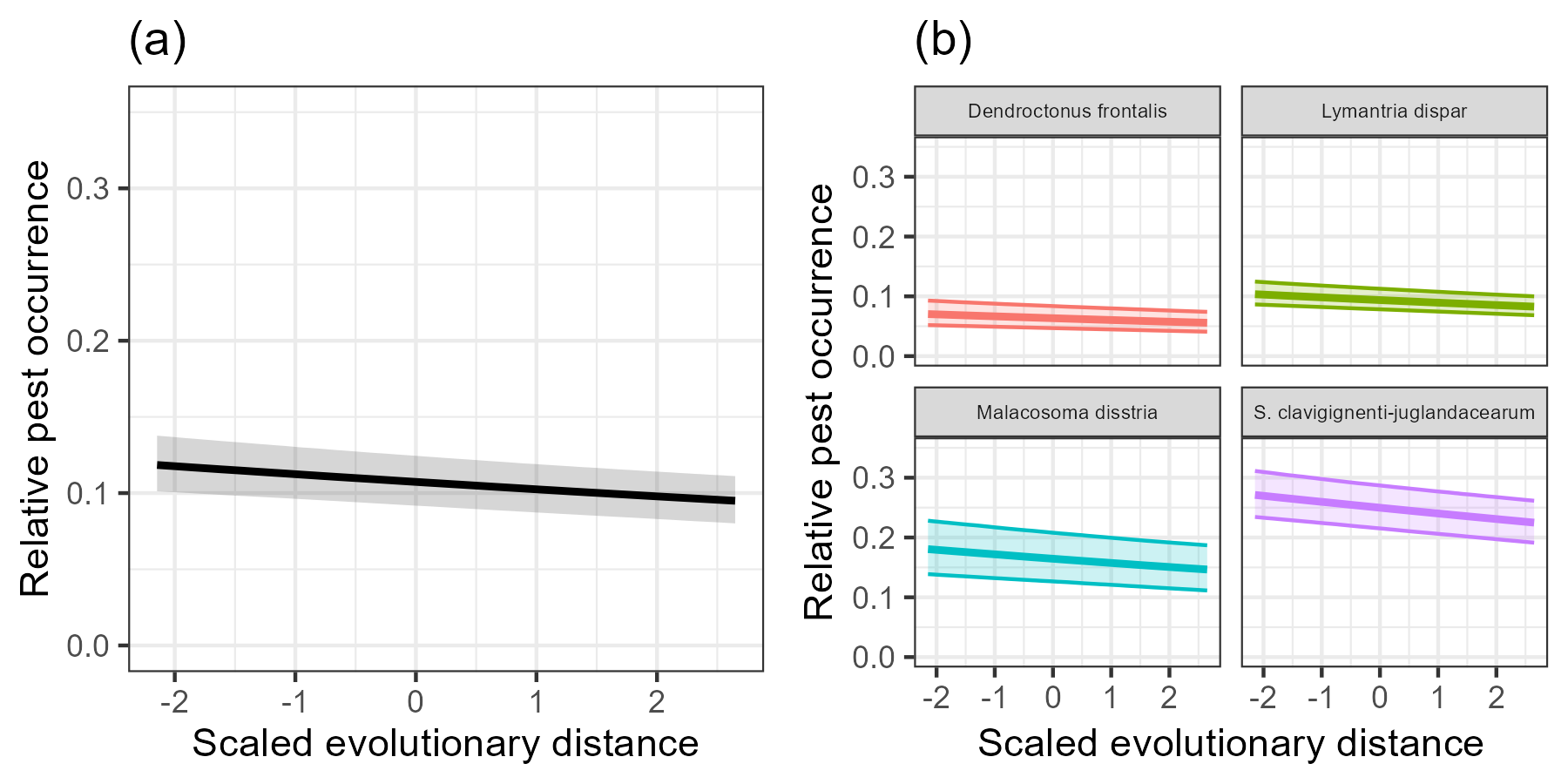

Supplement: S5 Fig — In general, S. clavigignenti-juglandacearum and M. disstria were the pests most diluted by tree diversity, while L. dispar and D. frontalis were minimally affected by tree diversity (see Figs 3 and 4). Data underlying this figure can be found at doi.org/10.6084/m9.figshare.21317979. (TIFF) [file pbio.3002473.s005.tiff]
