# Supplementary material for: Evolutionary history of host trees amplifies the dilution effect of biodiversity on forest pests
Source: PLoS Biol. 2024 Feb 27;22(2):e3002473. doi: 10.1371/journal.pbio.3002473 (PMC10898760; doi:10.1371/journal.pbio.3002473)
Supplement: Attachment — Submitted filename: responseToReviewers-fiaDilution.docx [file pbio.3002473.s007.docx]

REVIEWERS' COMMENTS:

Reviewer #1:

General comments. This paper presents a nice system for evaluating relationships between disease and host communities. The finding that evolutionary relationships help predict the dilution effect is not surprising, but welcome. Indeed it is at odds with popular conclusions in mammal communities that tend to focus on competence rather than relatedness. However, the study has flaws in logic, design and interpretation similar to many dilution effect studies. Some of these can be avoided with improvements to the analysis and more careful interpretation.

**We thank the reviewer for their positive comments and constructive feedback, which we have addressed in our revised submission, as we describe in more detail below.**

Specific comments.

119. An odd feature to the data are that only three damaging agents per tree are reported…What would happen if there were 7 damaging agents? Which agents are included? How does the exclusion affect your results? Looking at your data it seems that >3 agents would be rare, but with the prevalence data and some assumptions about independent assortment, you could get close to assessing how commonly this was the case and then argue why it is not likely to be a problem.

**We apologize that this was not clear in the original submission. Damaging agents are recorded as the surveyors assess the tree from the base of the tree to the canopy. The three most important damaging agents are then recorded for the tree. If a hypothetical tree had seven damaging agents, only the three most damaging would be recorded. The vast majority of trees we assessed, however, had two or fewer reported damaging agents which implies that most trees were not saturated with damaging agents. Randolph et al. (**[**https://doi.org/10.1007/s10661-020-08796-z**](https://doi.org/10.1007/s10661-020-08796-z)**) previously analyzed the frequency of tree species with three damages recorded, and found, on average across 20 tree species, ~1.5% of trees had three damaging agents. Although this percentage varied by tree species, it suggests very few trees had relevant damage that were not included in our model. We now include this additional information in the manuscript. See lines 125-130, which now read:**

**“Although some trees could, hypothetically, have been subject to more than three damaging agents, Randolph et al. [14] previously reported that very few individual trees (typically 1-2% across species) had three reported damaging agents, and an even smaller fraction (<1%) would be subject to four or more damaging agents. These data thus include the relevant damage for the vast majority of trees within the FIA sampling scheme.”**

Line 123. It is not enough to cite other sources for the limitations of the data. The limitations relative to your study are critical to present to the readers of this paper.

**Thanks for this suggestion. We have now summarized the limitations of the dataset highlighted in the reference and those we identified when conducting our analyses. See Lines 131-147.**

135: If non-host damage is treated as error, how frequent were such errors? What do they say about the dataset in general?

**Damage on non-documented hosts were infrequent, occurring on ~1% of the trees with a pest included in our analyses (1076 trees of 107108 trees with pest damage considered). In addition to acting as quality assurance for the host-associations observed in the dataset, removing non-host damage also had the secondary benefit of reducing spatial errors in the dataset. For instance, in the original dataset, white pine blister rust (*Cronartium ribicola*) was recorded in the southern US on various southern oak and pine species - despite this pathogen not being known in this area or on these specific hosts. More likely, this pathogen was confused with another rust pathogen (possibly *Cronartium quercuum*) which is frequent in the area, produces similar types of symptoms, and is known to occur on oaks and pines. More generally, errors in large databases such as FIA data are not uncommon, but by placing geographic and host constraints on pest occurrences helped ensure a higher quality dataset. We would be willing to re-run the models with these, potentially incorrect, occurrences, if the reviewer thinks this is important, but note we would have less confidence in the results of these models. See Lines 153-154, which reads:**

**“In total, this step removed approximately 1% of the attacked trees in the dataset from the pest prevalence calculation (1,076 trees of the 107,108 originally included).”**

140: how confident are you in the host range assessments from the literature? These are often highly subject to sampling error and bias. Why not use host range assessments from the data set instead? I would have more faith in those and they seem more relevant to the study. At the least, do it both ways.

**This is an important point. We invested large effort into compiling robust estimates of pest host range from a variety of independent sources. We used host information from outside the dataset for multiple reasons. First, by using independent sources of host ranges, we are able to use these as a quality check of the reliability of the host-pest associations reported in the dataset - as described above. Removing spurious host-pest associations had the additional benefit of removing pest occurrences in areas where they are not known to occur. Second, because the pest occurrences did not necessarily cover the entire geographic ranges of the pests, it is unlikely that the complete host range ranges could be derived from the FIA dataset alone. Spongy moth (*Lymantria dispar*), for instance, is known on hundreds of hosts and it is unlikely that all of these hosts would be captured in a single temporal snapshot (i.e., the most recent FIA sampling period used in our models). Further a comprehensive estimate of host range is needed for several of the metrics we include in the models - specifically the evolutionary distance metric and the total number of host species (as a metric of generalism or specialism). In total, removing hosts that were not known from other sources decreased the total number of attacked trees by approximately 1% (106,032 were kept of the original 107,108 trees attacked by the pests we considered). We’ve now added this detail to the manuscript. As above, we can re-run the model without this additional quality check, but we would feel less confident in the results of these models.**

Line 150. Incidence or prevalence. Incidence implies a per-time aspect of infection. I think you mean prevalence.

**In the plant pathology literature, incidence does not necessarily imply a temporal component as it does in the animal or medical pathology literature. Bock et al. (**[**https://doi.org/10.1007/s40858-021-00454-0**](https://doi.org/10.1007/s40858-021-00454-0)**), in their “phytopathometry glossary,” define disease incidence as “*the proportion of specimens diseased (regardless of the method) of the total number of specimens assessed (in other branches of science including medicine and entomology, this equates to prevalence. See definition of prevalence as used in plant pathology).*” However, we appreciate the potential for confusion and that we added to this confusion by using multiple terms to refer to the same metric, so we have switched all instances to “pest prevalence” for consistency, and include this definition in the manuscript. See 172-177.**

**“…we modeled pest prevalence as the number of attacked hosts per pest within plots, divided by the total number of trees within the plot. This metric aligns with the definition of pathogen ‘incidence’ in the plant pathology literature, and ‘prevalence’ in entomology and animal pathology literature [30]. For simplicity, we refer to this metric as ‘prevalence’, although we appreciate that there are other definitions of pathogen prevalence in the literature.”**

Line 150. I am all for Bayesian hierarchical models when applied to nested data (and Stan is the right choice), but Stan is hard for others to reproduce and you need to justify why you have used them. Also, why was an SEM approach not used? Finally, it is essential that others be able to reproduce your results. I see there is a promise to provide some of the data through Figshare and that other data are publicly available. Having writing a fair amount of R-Stan code, I know that links to data are not enough to reproduce the results. So please provide clear access to the data and annotated code used to produce the figures / key results. An R-Markdown file is an efficient way to do this.

**The data used in the models should already be available at the Figshare link provided in the manuscript, should the reviewer wish to review it. We have now included the R code to recreate the model and reproduce the main figures. We also now include a SEM in the manuscript (model shown in new figure S2), which includes, as suggested, an effect between diversity and host abundance. The directionality of most of the main effects are similar to those in the Bayesian model included in the manuscript. We describe the SEM in the Methods, but maintain the focus of the Results and Discussion on the Bayesian model, as it offers greater inference about how the dilution effect varies by individual pest species. We believe this latter component of the Bayesian model is critical as a major take-home message of our analyses is that the magnitude of dilution varies by pest, and we are able to quantify this variability in our Bayesian model by including pest-level predictors (e.g. pest generalism/specialism and forest community phylogenetic distance to a pest’s known host range, random effects of pest identity) that are not easily incorporated into the SEM.**

Line 153, 204 and 240. Although the dilution effect literature largely ignores it, it is critical to separate the effects of abundance from diversity. Any model trying to explain disease prevalence should include host density as the primary hypothesized driver. Residual effects of diversity may indicate a dilution effect. Furthermore, in an SEM, there may be indirect effects of density on diversity making the two difficult to separate. Line 204 implies you considered tree abundance as a driver, but it is critical to separate direct effects of abundance from diversity as well as the indirect effects of diversity on abundance. It is not entirely clear to me what was done. In my view, an SEM would be the right approach. To that end, to understand the diversity dilution effect, most models require assessing the assumption that communities are additive or substitutive. Dilution is more likely in substitutive communities. Given that your paper aims to explain variation in the dilution effect among sites, I would suggest that it is necessary to consider variation in additive vs substitutive effects among forest types. In short, please clarify the additive vs substitutive characteristics of the study system and carefully separate out the direct and indirect effects of density from "diversity".

**To clarify, total tree abundance within plots was accounted for in the response and explanatory variables. Pest prevalence was calculated as: number of trees attacked by a pest / total number of trees within the plot. Relative host abundance within the plot was included as an explanatory variable as: number of hosts of a pest in a plot / total number of trees within the plot. In the new SEM included in the manuscript, we allow for an effect between diversity and relative host abundance. Despite accounting for this effect, the main effects on pest prevalence are similar to those inferred from the previous Bayesian model.**

**Regarding additive vs. substitutive effects among forest types, shifts in forest composition away from hosts utilized by pests should be captured by our abundance-weighted metric of evolutionary distance. Our metric presumes that, for a pest that specializes on pines, replacing one pine with another would have a smaller effect on prevalence than replacing a pine with an angiosperm. As such, the effect of forest composition is not uniform across pests, but rather dependent on the hosts utilized by the pest. By including this metric, along with Shannon diversity, and the proportion of trees that were known hosts, we can separate the effects of total diversity (Shannon; we note that species richness was highly correlated with Shannon diversity, *r* = 0.88) from shifts in composition (proportion of hosts and evolutionary distance). We apologize if this was not sufficiently clear in our original submission, we certainly agree with the reviewer that these are critical components of our analysis.**

Line 160. There are many diversity metrics. And they measure different things and often generate different results. Past dilution studies have cherry picked metrics that fit their expectations rather than those that fit predictions from theory. Justify your choices of metrics better. And, indicate to the reader how your results would have differed if you had used other common metrics (and why).

**We chose Shannon diversity metric as it includes information about both tree richness and abundance - both of which we expect to affect pest prevalence - and we show it to be a good predictor in our model. We consider this metric preferable to species richness, frequently used in dilution studies, which may not adequately represent diversity when there is large variability in species abundances. We calculated additional diversity metrics, Simpson's index, inverse Simpson, and species richness, and found them to be strongly correlated with Shannon diversity used in our analyses (*r* = 0.96, 0.92, 0.88, respectively), indicating that they contain much of the same information. We now include this information in Methods of the manuscript. It would, of course, be possible to run our models substituting Shannon diversity for any one of the numerous alternative diversity metrics; however, we do not believe they would materially improve model fit. See Lines 186-188, which reads:**

**“We also examined Simpson’s index, inverse Simpson’s, and species richness, but found them all strongly correlated with Shannon diversity (*r* = 0.96, 0.92, 0.88), indicating that these metrics contain much of the same information.”**

WRT metrics, you should also consider pathogen diversity as a function of forest diversity. The predictions there are pretty straightforward (host diversity begets parasite diversity). It would be easy for you to assess this association with your data at the plot level.

**Unfortunately, the available data from the FIA is unsuitable for tabulating pest diversity, as the pests we assessed (*n* = 60) represent only a small fraction of all pests in US forests. Further, the vast majority of plots (~98%) only have one or two recorded species-specific pests, and an initial test of the correlation between tree and pest richness revealed only a weak relationship (*r* = 0.07). While we agree this is an interesting question to address, better, more comprehensive, pest occurrence data is likely required to adequately address it.**

Line 162. BTW, this is a great way to express "diversity" (far better than species counts). You might want to highlight this distinction for the reader as well as the motivation for using it and how it applies (and does not apply to the dilution effect). In doing so, think carefully about evolutionary distance at different taxonomic groupings. E.g., ferns and trees are really different. And a forest with a lot of ferns would be more diverse. But you don't consider ferns because you focus only on tree health. This is just to say that your "diversity" is limited in scope. Explain why and how it might affect your results.

**We thank the reviewer for this suggestion, and now include additional text regarding why the phylogenetic relatedness between pest hosts and forest composition can affect pest occurrences. The reviewer raises an important point that the evolutionary distance metric is sensitive to how forest composition is defined (i.e, whether trees, shrubs, herbaceous plants, etc. are included). In our case we focus on tree pests, and most identified pests primarily affect only trees, we thus calculate our phylogenetic distance only among trees. As the FIA plots are concentrated in forest ecosystems where trees are the dominant plant type, we likely encompass most of the plant biomass within plots. However, we have included a caveat in the manuscript stating that the evolutionary distance metric is partially contingent on how the host community is defined (e.g., trees, shrubs, etc.), and that using a broader definition of forest community composition could be particularly useful for generalist pests not limited to trees. See lines 193-198, which reads:**

**“The phylogenetic composition of host communities has been shown to be an important constraint on pest prevalence [12]. We here consider only the tree community composition as our focus is on pests that primarily affect tree species; however, for more generalist pests that can infest a variety of plant types (e.g., trees, understory shrubs, and herbaceous plants), it would be possible to expand this metric of evolutionary diversity to capture other elements of the plant community.”**

Line 169. I agree that is it good to control for climate, but latitude and altitude are also possible joint drivers of disease and forest diversity. These variables must be considered as drivers, ideally through an SEM framework.

**Thanks for this suggestion. We agree that pest prevalence can be affected by elevation and latitude, but we found both to be very strongly correlated with average temperature (*r* between latitude and temperature = -0.93; *r* between log elevation (+4) and temperature = -0.78), so we chose not to include these variables in the models. Given the high correlation between these variables, it is unlikely that elevation and latitude capture much additional information on pest prevalence not already captured by the current suite of included predictors.**

Line 171. I would not consider -.61 to be a weak covariance. Moderate perhaps.

**Agree, this has been changed.**

Line 176. If I understand correctly, you are considering the effect of tree diversity on the prevalence of a particular pest. Please also consider the effect of tree diversity on the combined prevalence of all pests. This is critically important if you want to discuss how diversity affects forest health (which is the effect of all pests combined, not the average effect of each pest individually).

**Our modelling framework allows us to infer both the average effect (accounting for all pests species) and the average effect of each pest species individually. The average fixed effect, accounting for all pests, indicates a tendency toward dilution. This result is shown in Figure 2, which shows the posterior distribution of the parameter estimates. The combined effect of diversity on prevalence is also now captured in the SEM, which does not account for individual pest differences, but likewise indicates a negative relationship.**

Line 271. I think this grossly oversimplifies the Phytophtora story. It is an important system to discuss, but evidence for dilution is strongly sensitive to how one measures diversity. Please look into this a bit more.

**We apologize for oversimplifying this example, and we have added additional details about dilution and *P. ramorum*. In using this example, we are mainly trying to highlight that a generalist pathogen can be diluted by forest biodiversity, depending on the biology of the system. We now include the more recent Rosenthal paper, and mention that dilution can vary among the hosts of the individual pathogen. See lines 317-323, which reads:**

**“*Phytophthora ramorum*, a generalist pathogen and causal agent of Sudden Oak Death, for instance, has been shown to be diluted by diversity [43]. Importantly, however, while a dilution effect may be observed at the community level, the probability of particular host species being infected by *P*. *ramorum* may be independent of diversity or even show the opposite relationship – being amplified by diversity rather than diluted [44]. This may be the case when a single, highly competent host tree dominates pest dynamics in the community.”**

Line 275. Although you might not have information about competence, you likely do have information on growth rates. Most dilution literature in the animal system speaks to the following: low diversity systems have weedy competent hosts, whereas diverse systems have more well-defended long lived hosts. I.e., in animal systems, the current views are more about correlations between diversity and competence rather than on diversity per se. I don't necessarily agree that this is a rule, but it could be measured in your system. Please explore as this explanation competes with your interpretation of evolutionary distance.

**We agree there are likely additional community-level metrics that could affect pest prevalence, including the proportion of weedy vs. slow-growing species in plots. While we expect some of these sorts of metrics to be correlated with our evolutionary distance metric, which accounts for both the identity and abundance of species in the forest plots, including additional functional data could improve our understanding of pest prevalence. As suggested we have included additional text regarding community-level effects that may explain pest prevalence. See lines 324-334, which reads:**

**“In addition to information on host competence, other community-level data could help improve our understanding of pest prevalence. Functional traits have been linked to total pest loads and, when aggregated to the community, could affect the likelihood of pests establishing in a forest. For example, the proportion of fast-growing weedy species, that trade defense for growth, may be more likely to amplify pests compared to forests made up of slow-growing species that invest in various defense strategies. Total forest productivity, leaf area, or functional type (angiosperm vs. gymnosperm), amongst other forest characteristics, could each affect forest susceptibility to pest attack. Many such traits are phylogenetically conserved so could be partially captured by our evolutionary distance metric, but future work identifying landscape-level effects could be particularly informative for understanding pest prevalence and how they may shift with future landscape change.”**

Line 285. Thanks for bringing up the pine-oak rust example. It is a nice example of how complex life cycle parasites might have different relationships to diversity. Yet your data do not consider this variable. Why not?

**In general, this sort of information on lifecycle complexity is unavailable, and although we agree that it is likely important, it is not always clear how this complexity should be quantified, or reconciled between insect pests and pathogens. For example, lifecycle complexity can vary by host associations, even for individual pests. The pine-oak rust, for instance, can perpetuate on oaks, but infection of pine requires an oak. More detailed studies on particular pests or pathogens could improve our understanding of how lifecycle complexity affects pathogen occurrences on individual hosts. We have highlighted this challenge with additional text in this section. See Lines 341-347, which reads:**

**“These dynamics, however, can be complicated by pests’ lifecycle. Pests that rely on a particular sequence of hosts for successful infection (e.g., heteroecious rusts), or when transmission rates between hosts are not uniform (for instance when insect vectors have feeding preferences and vary in their abilities to transmit pathogens [52]), may respond differently to diversity than those pests without strict host requirements. In these cases, pests response to diversity may be more impacted by host co-occurrence and, potentially, their interactions.”**

Line 299. This speculation is common in dilution effect studies. It is part of the negativity bias in conservation biology and is logically flawed. For instance, pathogen prevalence is not necessarily related to extinction risk. That greatly depends on the pathogen and host. And one could argue that a higher pathogen prevalence in low diversity stands is a mechanism that helps maintain forest diversity (Janzen-Connell hypothesis). For your concluding paragraph to be supported, you would have to show that rare species suffer higher impacts from pathogens in low diversity forests. I encourage you to investigate and report on this particular potential for feedbacks between pathogens and host diversity in your data. Otherwise, please avoid the negativity bias.

**Unfortunately, as discussed above, the FIA data is inadequate to directly address the link between host diversity and pathogen diversity. We have moderated our language here to emphasize the complex effects pests can have on forest diversity - from elevating extinction risk to enhancing forest diversity - depending on both the pest, its hosts, and the composition of the forest where they occur.**

Reviewer #2:

The authors present the results of an analysis of the effects of a suite of variables on the occurrence of pests and pathogens of trees, using a database of tree health and composition available from the US Forest Service. They find strong evidence for dilution effects for many pests/pathogens, with Shannon diversity reducing prevalence, temperature increasing prevalence, and average phylogenetic distance of trees from the preferred host decreasing prevalence.

This is a generally well-written manuscript that utilizes a comprehensive database of forest pests and pathogens to address an important concept. The magnitude of the data is excellent - dozens of pests and pathogens from thousands of forest locations. Generally, I suggest that the authors make the work more scholarly, particularly around their exploration of prior work on dilution effects. That said, my comments are addressable. This paper is phenomenological rather than mechanistic, and the authors could expand their exploration of potential underlying mechanisms.

**We thank the reviewer for their feedback. We acknowledge that our paper is primarily an exploration of the diluting effect of biodiversity on pest prevalence – and much of its strength comes from the size and breadth of the dataset we analyse. However, the form of our model is designed to explicitly test well-developed hypothesis in the disease ecology and forest pathology literature on the diluting effects of biodiversity, and all model predictors have clear, interpretable contributions, that can be related back to underlying mechanisms thought important in driving forest pest prevalence.**

One major area that could be improved during revision is on the response variable. I believe they are using the percent of trees infected, which would be prevalence, but they are not entirely clear on this point. E.g. in the abstract, they refer to "pest occurrences." In the Methods, e.g. line 150ish, they say that they modeled pest incidence, as the number of attacked hosts per pest within plots, standardized by total tree frequency. I'm not sure I could recreate this value from this information. How did they standardize total tree frequency? This is important because Rosenthal et al. and others have found a decline in percentage of infected hosts, while getting a different result if non-hosts are included.

**We apologize that this was not clearer in our original submission. The metric is calculated as: number of trees attacked by a given pest within the plot / number of trees within the plot. This aligns with the definition of “incidence” in the plant pathology literature - Bock et al. (**[**https://doi.org/10.1007/s40858-021-00454-0**](https://doi.org/10.1007/s40858-021-00454-0)**), in their “phytopathometry glossary,” define disease incidence as “*the proportion of specimens diseased (regardless of the method) of the total number of specimens assessed ...*” We have clarified this in the manuscript, and adjusted the terminology throughout the manuscript for consistency. See lines 172-177, which reads:**

**“… we modeled pest prevalence as the number of attacked hosts per pest within plots, divided by the total number of trees within the plot. This metric aligns with the definition of pathogen ‘incidence’ in the plant pathology literature, and ‘prevalence’ in entomology and animal pathology literature [30]. For simplicity, we refer to this metric as ‘prevalence’, although we appreciate that there are other definitions of pathogen prevalence in the literature.”**

**In addition, we now also discuss how dilution can occur at the community level but may not always be apparent at the level of individual hosts trees, as in the example Rosenthal et al. discuss, where a single highly competent host tree can drive pest dynamics (see lines 319-323).**

The authors intermingle results for pests (e.g. insects) and pathogens (e.g. viruses and fungi) here, without making distinction between them. Some prior work has been done looking at this distinction, I believe by Dave Civitello, and they should explore this here.

**We thank the Reviewer for this suggestion. In our Bayesian model, we include pest identity as a random effect and pest type as a factor in our model to capture their unique contributions (see Table S1). We now also include a new figure (Fig S3.) that shows pests, identified by broad taxonomic grouping (e.g. insect, pathogen, parasitic plant), along the dilution and host abundance gradients. This figures helps illustrate that pathogens, insects, and parasitic plants are interspersed along these axes.**

Line 63 - "There is" could be rewritten to make a stronger start, e.g. "Better understanding the role of biodiversity in xxx is an urgent need."

**Done. We thank the reviewer for the suggestion.**

Line 70 - This isn't accurate. All recent meta-analyses I can think of have supported the overall presence of dilution effects, whether for plants or animals. Authors of these meta-analyses often focus on their subtle differences, e.g. stronger for fungal pathogens than viral ones, or stronger for temperate than tropical systems. But there is, unequivocally, consensus that there are dilution effects and that they often occur. The three citations the authors indicate here are not formal meta-analyses, and at least one of them (Lafferty and Wood) is decidedly out of date in this fast-moving field. Their claims in this section are critically important to their paper. I encourage them to be more scholarly here, by citing the recent overview of the dilution effect by Ostfeld and Keesing for eample, and also by focusing on meta-analyses rather than narrative explorations of evidence. Here, they cite one paper about the diversity of managed forests from 2006. In this section, they should cite general (current) literature on dilution effects. If they then want to specialize on results from forests, they should do so more thoroughly. For example, there's a recent paper by Lisa Rosenthal that is relevant to their results, and they should explore the literature on forest pests/pathogens in real detail once they establish the general support for dilution. Also, generally, it's fine to say that dilution effects don't always occur, but not accurate to say we don't know when they will or won't - at this point there are lots of studies that show them stronger in some types of systems than others, etc.

**We appreciate the reviewer’s feedback here, and we acknowledge that we perhaps provided a somewhat skewed perspective by looking to highlight and contrast previous work that reported conflicting patterns, while understating the growing consensus in the more recent literature. We have, therefore, gone back to the primary literature to ensure that we provide a more accurate summary of recent work and a better summary of the current consensus on the dilution effect, particularly in forests. We have now revised this section (see lines 70-75) and include additional citations on dilution effects in forests, including Guyot et al. 2016, Hantsch et al 2014, Wein et al. 2016, amongst others. We also cite the 2021 Rosenthal paper on line 319-322. Finally, we also recognize that there is now a much greater understanding of how the magnitude of the dilution effect varies across different systems; however, we** **still lack robust generalizable models that allow us to make predictions across less well-studied pest systems that also account for potentially confounding variables– the goal of our analysis here.**

Line 76 - Start with something other than "It is likely…", e.g. "Tree pest responses are likely influenced…"

**Done**

Line 195 - rephrase for clarity: "Infestation with a particular pest was reported on average (median) in 99 (±1140.56 SD) of the total 26,343 plots."

**Done. See Line 240.**

Line 236 - Here, some references supporting the statement would be useful. Also, the "but" here is strange because the Liu et al. paper strongly supports dilution effects across plants, based on their meta-analysis. I assume the "but" is because they find that dilution is stronger in some instances than others, but that's a subtle point here that either needs to be explored in more detail or omitted in favor of the overall finding of their study.

**The “but” here is included because Liu show no general effect of dilution in forest ecosystems (i.e., the focus here), which we now emphasize. The authors suggest this is due to differences in the importance of host identity and characteristics in forests versus in grasslands (where the authors found strong dilution effects). We recognize this is a subtle point, and so have clarified this in the parentheses here.**

Line 238 - Here, cite references supporting the claim about host abundance. I assume they mean Rosenthal et al.?

**We have included citations here that specifically discuss the interplay between abundance and diversity, including Keesing & Ostfeld 2021, and Jactel et al. 2021**

Figure 3 - Here, "bolder lines" is confusing. Please edit to say shaded segments, or something like that.

**Done**

Figure S3 - In this figure, panel a has a non-zero y-axis, which dramatizes the effect, but which is also somewhat misleading. It should be re-drawn with zero, as in panel b.

**We have remade this figure so that each panel includes the same axes, and includes zero on the y-axis.**
