# Supplementary material for: Evolutionary history of host trees amplifies the dilution effect of biodiversity on forest pests
Source: PLoS Biol. 2024 Feb 27;22(2):e3002473. doi: 10.1371/journal.pbio.3002473 (PMC10898760; doi:10.1371/journal.pbio.3002473)
Supplement: Attachment — Submitted filename: response.docx [file pbio.3002473.s008.docx]

IMPORTANT - Please address the following:

a) Please could you change your Title to something more explicit for our broader readership? We suggest "The evolutionary history of host trees amplifies the dilution effect of biodiversity on forest pests," which clarifies the nature of the organisms studied.

**Done**

b) Please provide a blurb, according to the instructions in the submission form.

**We now include a blurb in the submission form.**

c) Please address my Data Policy requests below; specifically, we need you to supply the numerical values underlying Figs 1, 2, 3, 4, S1AB, S3, S4, S5AB, either as a supplementary data file or as a permanent DOI’d deposition, perhaps as part of your existing Figshare deposition.

**We now include all of the data and code underlying the figures at the Figshare repository, available at doi.org/10.6084/m9.figshare.21317979**

d) Please cite the location of the data clearly in all relevant main and supplementary Figure legends, e.g. “The data underlying this Figure can be found in S1 Data” or “The data underlying this Figure can be found in <https://gcc02.safelinks.protection.outlook.com/?url=https%3A%2F%2Ffigshare.com%2Fs%2F56925a31df28fce26562%25E2%2580%259D&data=05%7C01%7Candrew.gougherty%40usda.gov%7C22de0db78c784a2f6d3f08dbf6866260%7Ced5b36e701ee4ebc867ee03cfa0d4697%7C1%7C0%7C638374829260819442%7CUnknown%7CTWFpbGZsb3d8eyJWIjoiMC4wLjAwMDAiLCJQIjoiV2luMzIiLCJBTiI6Ik1haWwiLCJXVCI6Mn0%3D%7C3000%7C%7C%7C&sdata=W3tz0qeGugmyZ8h5Mz1hVbRBqoTbvcMU0z4oCNtnnnI%3D&reserved=0>

**We’ve included similar text in all the figure legends.**

e) Please make any custom code available, either as a supplementary file or as part of your Figshare deposition (I see that the latter already contains R script for model fitting).

**We’ve included code for the model, subsequent analyses and figures in the Figshare repository.**

As you address these items, please take this last chance to review your reference list to ensure that it is complete and correct. If you have cited papers that have been retracted, please include the rationale for doing so in the manuscript text, or remove these references and replace them with relevant current references. Any changes to the reference list should be mentioned in the cover letter that accompanies your revised manuscript.

**To our knowledge, none of the references have been retracted, nor have we made any changes to the references.**

We expect to receive your revised manuscript within two weeks.
